# Intranasal Delivery of Inactivated Influenza Virus and Poly(I:C) Adsorbed Corn-Based Nanoparticle Vaccine Elicited Robust Antigen-Specific Cell-Mediated Immune Responses in Maternal Antibody Positive Nursery Pigs

**DOI:** 10.3389/fimmu.2020.596964

**Published:** 2020-12-16

**Authors:** Veerupaxagouda Patil, Sankar Renu, Ninoshkaly Feliciano-Ruiz, Yi Han, Anikethana Ramesh, Jennifer Schrock, Santosh Dhakal, Harm HogenEsch, Gourapura J. Renukaradhya

**Affiliations:** ^1^ Food Animal Health Research Program, Ohio Agricultural Research and Development Center, College of Food, Agricultural and Environmental Sciences, Wooster, OH, United States; ^2^ Department of Veterinary Preventive Medicine, College of Veterinary Medicine, The Ohio State University, Columbus, OH, United States; ^3^ Department of Comparative Pathobiology, College of Veterinary Medicine, Purdue University, West Lafayette, IN, United States

**Keywords:** inactivated SwIAV, corn-based nanovaccine, cross-protective cell meditated immunity, polyfunctional T cells, maternal antibody, Poly(I:C), intranasal route, maternally derived antibody positive pigs

## Abstract

We designed the killed swine influenza A virus (SwIAV) H1N2 antigen (KAg) with polyriboinosinic:polyribocytidylic acid [(Poly(I:C)] adsorbed corn-derived Nano-11 particle based nanovaccine called Nano-11-KAg+Poly(I:C), and evaluated its immune correlates in maternally derived antibody (MDA)-positive pigs against a heterologous H1N1 SwIAV infection. Immunologically, in tracheobronchial lymph nodes (TBLN) detected enhanced H1N2-specific cytotoxic T-lymphocytes (CTLs) in Nano-11-KAg+Poly(I:C) vaccinates, and in commercial vaccinates detected CTLs with mainly IL-17A^+^ and early effector phenotypes specific to both H1N2 and H1N1 SwAIV. In commercial vaccinates, activated H1N2- and H1N1-specific IFNγ^+^&TNFα^+^, IL-17A^+^ and central memory T-helper/Memory cells, and in Nano-11-KAg+Poly(I:C) vaccinates H1N2-specific central memory, IFNγ^+^ and IFNγ^+^&TNFα^+^, and H1N1-specific IL-17A^+^ T-helper/Memory cells were observed. Systemically, Nano-11-KAg+Poly(I:C) vaccine augmented H1N2-specific IFNγ^+^ CTLs and H1N1-specific IFNγ^+^ T-helper/Memory cells, and commercial vaccine boosted H1N2- specific early effector CTLs and H1N1-specific IFNγ^+^&TNFα^+^ CTLs, as well as H1N2- and H1N1-specific T-helper/Memory cells with central memory, IFNγ^+^&TNFα^+^, and IL-17A^+^ phenotypes. Remarkably, commercial vaccine induced an increase in H1N1-specific T-helper cells in TBLN and naive T-helper cells in both TBLN and peripheral blood mononuclear cells (PBMCs), while H1N1- and H1N2-specific only T-helper cells were augmented in Nano-11-KAg+Poly(I:C) vaccinates in both TBLN and PBMCs. Furthermore, the Nano-11-KAg+Poly(I:C) vaccine stimulated robust cross-reactive IgG and secretory IgA (SIgA) responses in lungs, while the commercial vaccine elicited high levels of serum and lung IgG and serum hemagglutination inhibition (HI) titers. In conclusion, despite vast genetic difference (77% in HA gene identity) between the vaccine H1N2 and H1N1 challenge viruses in Nano-11-KAg+Poly(I:C) vaccinates, compared to over 95% identity between H1N1 of commercial vaccine and challenge viruses, the virus load and macroscopic lesions in the lungs of both types of vaccinates were comparable, but the Nano-11-KAg+Poly(I:C) vaccine cleared the virus from the nasal passage better. These data suggested the important role played by Nano-11 and Poly(I:C) in the induction of polyfunctional, cross-protective cell-mediated immunity against SwIAV in MDA-positive pigs.

## Introduction

Swine influenza A virus (SwIAV) constitutes a significant economic and health risk to both animals and humans throughout the world. Pigs by virtue of their susceptibility to both avian and mammalian influenza A viruses, represent an intermediate animal reservoir facilitating genetic reassortment and interspecies dissemination. Currently, three circulating SwIAV subtypes (H1N1, H1N2 and H3N2) have been identified in both swine herds and human populations. Hence, the reduction of the rate of SwIAV infections in domestic pigs is crucial (1). This objective can be realized by use of efficacious vaccines, which confer broadly cross-protective and long-lasting immunity against evolving strains and subtypes even in the presence of MDA in finisher pigs. The persistence and levels of MDA in finisher pigs are highly variable resulting in variations in the response to SwIAV vaccines and high risk of seasonal SwIAV outbreaks. Current injectable SwIAV vaccines are plagued by several deficiencies such as the lack of cross-protective immunity, inability to elicit mucosal secretory IgA (SIgA) responses, and susceptibility to interference by MDA (2). Efforts are being made to overcome the problem of interference of MDA with mucosal vaccines (3, 4). In these studies, vaccines are delivered intranasally to target the nasal/nasopharynx-associated lymphoid tissues (NALT) leading to the induction of strong cognate immune responses (5–7). Intranasal administration of vaccines against respiratory pathogens is performed to simulate natural infection leading to a robust induction of antigen-specific IgA and cell-mediated immune responses. This is attributed to the induction of antigen-specific mucosal immune responses in the NALT (6, 8). However, the induction of effective immune responses following intranasal vaccination is challenging because of the tolerogenic mucosal environment. Furthermore, since the SwIAV KAg lacks adequate antigenicity, potent adjuvants such as Poly(I:C) can boost the antigen-specific antiviral responses (9, 10). Hence, there is an urgent unmet demand for novel intranasal vaccines that counter the current threat of SwIAV more effectively even in the presence of MDA.

Nanoparticles (NPs)-based vaccines [nanovaccines] are revolutionizing the field of vaccinology. Nanovaccines are advantageous over oil adjuvant-based inactivated virus and subunit vaccines. Nanovaccines can protect vaccine antigens from degradation leading to amplification of the bioavailability *via* slow release of antigens and targeting antigens to antigen- presenting cells such as dendritic cells (11). Furthermore, inactivated virus and subunit antigens can be fortified with adjuvants such as synthetic double-stranded RNA, toll-like receptor-3 (TLR-3) ligand, Poly(I:C) to elicit robust antigen-specific immune responses (12, 13). The KAg adjuvanted with Poly(I:C) upon intranasal delivery elicited a strong heterologous mucosal antibody response in pigs (14). Previously, our lab designed and characterized corn-based cationic alpha-D-glucan nanoparticles (Nano-11) and confirmed its potential as a reliable nanovaccine platform in pigs (15–18). Nano-11 has inherent immunostimulatory properties and acts as an adjuvant (15, 16). Hence, the present study was launched with an objective of developing a novel vaccine composed of KAg with Poly(I:C) co-adsorbed together on Nano-11 [Nano-11-KAg+Poly(I:C)] for use in MDA-positive pigs. We hypothesized that this vaccine offers protection even in the presence of MDA.

Influenza-specific cell-mediated immune responses play an important role in host defense mechanisms. Accumulating body of scientific data in humans, mice and pigs corroborate the critical protective role of antigen-specific cell-mediated immune responses. The protective role of cross-reactive CD8^+^ T-cells targeting conserved viral epitopes was reported by earlier pioneering studies (19). Likewise, the presence of antigen-specific T-cells led to the amelioration of symptoms and reduced shedding in case of 2009 influenza pandemic in humans (20, 21). In mice, influenza virus infection elicits antigen-specific T-cell responses leading to protection in the absence of neutralizing antibodies (22). Administration of signal minus Flu (S-FLU) universal influenza vaccine led to alleviation of lung pathology and virus burden in nasal passages and lungs following homologous and moderately matched virus challenge devoid of neutralizing antibodies in pigs (23). In addition, intranasal delivery of poly D,L-lactic-*co*-glycolic acid (PLGA) nanoparticles entrapped with H1N1 influenza virus derived conserved peptides elicited a robust antigen-specific T-cell response but not neutralizing antibody response leading to reduced lung pathology following a heterologous virus challenge in pigs (24). Furthermore, in pigs, the kinetics of SwIAV H1N2-specific cell-mediated immune responses were analyzed. In the lung draining TBLN at 4 days post-infection (DPI), proliferating CD4^+^ T-cells were identified (25). In addition, at 9 and 12 DPI, maximum frequencies of polyfunctional IFNγ^+^IL-2^+^TNFα^+^CD4^+^ T-cells were detected in both PBMCs and TBLN mononuclear cells (MNCs). In SwIAV infected pigs at 44 DPI, IFNγ^+^&TNFα^+^, IFNγ^+^&IL-2^+^, and TNFα^+^&IL-2^+^ CD4^+^ T-cells were present in the lungs, TBLN, and PBMC. The predominant frequencies of CD4^+^ T-cells, CD4^+^CD8^+^ T-cells in SwIAV infected pig bronchoalveolar lavage (BAL) has also been reported. However, H1N2-specific proliferating IFNγ^+^ or IFNγ^+^&TNFα^+^ double positive Perforin^+^CD27^+^CD8^+^ T-cells reached peak frequencies at 6 DPI and persisted up to 44 DPI in lungs (25). Following the H1N1 challenge, CD8^+^ T-cells translocated to lungs and BAL at 6 DPI (26). These findings emphasize the significance of antigen-specific polyfunctional cell-mediated immune responses. Therefore, in this study we characterized the array of SwIAV-specific cell-mediated immune responses in MDA-positive pigs, which received intranasal Nano-11-KAg+Poly(I:C) and compared vaccine efficacy with intramuscular commercial SwIAV vaccine.

## Methods

### Vaccines and Challenge Viruses

Inactivated SwIAV H1N2-OH10 (A/Swine/OH/FAH10-1/10) (27) was used in the Nano-11 based candidate vaccine and for challenge infection SwIAV H1N1-OH7 (A/Swine/OH/24366/2007) (28) was used. For determining the cross-protective antibody responses *in vitro*, a heterosubtypic IAV H3N2-OH4 (A/Turkey/OH/313053/2004) (29) was used in assays. Viruses were propagated in Madin-Darby canine kidney (MDCK) cells. The FluSure XP^®^ commercial inactivated SwIAV vaccine was purchased from Zoetis (MI, USA). It is a multivalent commercial inactivated vaccine comprising H1N1, H1N2, and H3N2 SwIAVs in an oil adjuvant. Nano-11 was prepared as previously described (16). The Nano-11 based vaccine formulation was prepared using KAg adsorbed with or without the TLR-3 agonist Poly(I:C) electrostatically on Nano-11 leading to two vaccine formulations, Nano-11-KAg+Poly(I:C) or Nano-11-KAg, respectively. Furthermore, one more formulation KAg+Poly(I:C) was prepared and used as a control (17). Poly(I:C) was procured (Invivogen, CA, USA) and dissolved as per the manufacturer’s instructions and aliquots were stored frozen.

### Animal Studies

Three-week old influenza MDA positive weaned pigs born to sows vaccinated with FluSure XP^®^ twice during pregnancy as per the manufacturer guidelines were procured and vaccinated intranasal through both the nostrils twice with Nano-11-KAg+Poly(I:C) or as a control Mock saline, KAg+Poly(I:C), Nano-11-KAg by using a spray mist delivery device (Prima Tech USA, NC) (30). The commercial vaccine was administered intramuscular as per manufacturer’s guidelines. Pigs were boosted with the same dose of vaccine and route after three weeks of prime vaccination. Blood was collected on day 34 post- prime vaccination (Prechallenge; PC) for the isolation of PBMCs in restimulation assays *in vitro*. Two weeks after the second dose of vaccination (Day 35), pigs were challenged with a virulent SwIAV SW/OH/24366/2007 (H1N1-OH7), 6 × 10^6^ TCID_50_/ml per pig 50% amount by intranasal and other 50% by intratracheal route (30). Pigs were kept under observation for clinical disease and euthanized at post-challenge day 6 (DPC6), examined the lungs, and collected nasal swabs, BAL fluid, lung, TBLN, and blood for immune and viral analyses (**Supplementary Figure 2**). Each of the above-mentioned experimental groups comprised of 4–6 pigs, and the number of animals that were used in the experimental analyses has been mentioned in the respective figure legends.

### Analysis of T Cell Populations

PBMCs isolated at day 34 post-prime vaccination (prechallenge) and DPC6 and TBLN mononuclear cells (MNCs) isolated at DPC6 were restimulated with H1N2-OH10 and H1N1-OH7 SwIAV KAg [10 ug/ml] for 72 hours (hr) *in vitro*. The cells were labeled and analyzed by flow cytometry for frequencies of cytotoxic T-lymphocyte (CTL) [CD3^+^CD4^-^CD8α^+^β^+^], T-helper/Memory cells [CD3^+^CD4^+^CD8α^+^β^-^], T-helper cells [CD3^+^CD4^+^CD8α^-^β^-^], and naïve T-helper cells [CD3^+^CD4^+^CD8α^-^β^-^CD27^+^] as described previously (17, 30).

### Surface and Intracellular Cytokine Staining for Flow Cytometry

**Figure 1:**
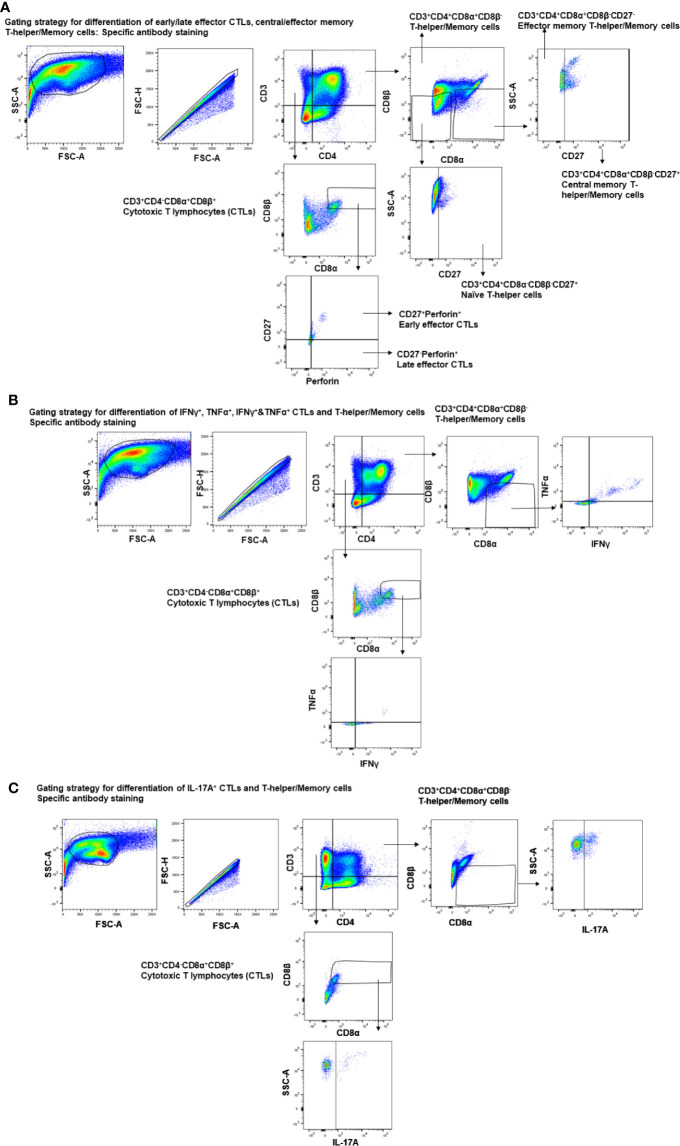
Gating strategy employed for the analysis of CTLs, T-helper/memory cells, total T-helper cells, and naïve T-helper cells. Three-week-old maternal antibody positive pigs were administered twice with Nano-11-KAg+Poly(I:C) or controls mock saline, KAg+Poly(I:C), Nano-11-KAg or commercial multivalent SwIAV vaccine and challenged at day post-prime vaccination 35 with H1N1-OH07 SwIAV. PBMCs isolated at day post-prime vaccination 34 (Prechallenge; PC) and DPC6 and TBLN MNCs isolated at DPC6 were stimulated with H1N2-OH10 or H1N1-OH7 SwIAV for 72 h *in vitro*. The cells were immunostained and analyzed by flow cytometry for the frequencies of different types of CTLs [CD3^+^CD4^-^CD8α^+^β^+^], T-helper/Memory cells [CD3^+^CD4^+^CD8α^+^β^-^], T-helper cells [CD3^+^CD4^+^CD8α^-^β^-^], and naïve T-helper cells [CD3^+^CD4^+^CD8α^-^β^-^CD27^+^] for **(A)** early/late effector CTLs, central/effector memory T-helper/Memory and naïve T-helper cells - specific antibody staining; **(B)** IFNγ^+^, TNFα^+^, IFNγ^+^&TNFα^+^ CTLs and T-helper/Memory cells - specific antibody staining; **(C)** IL-17A^+^ CTLs and T-helper/Memory cells–specific antibody staining.

Approximately 10 million PBMCs or TBLN MNCs isolated at indicated time points as described previously (30) and were seeded in a 24-well flat bottom plate in 2-ml total volume of culture medium (RPMI 1640, 10% FBS). The cells were restimulated with H1N2-OH10 SwIAV at 0.1 multiplicity of infection (MOI) and H1N1-OH7 SwIAV KAg at 10 ug/ml for 72 h *in vitro*. Protein transport inhibitors Brefeldin A (GolgiPlug) and Monensin (GolgiStop) (BD Biosciences, San Jose, CA) were added for the last 6 h of the incubation period. Cells were harvested, washed, blocked with 1% normal rabbit serum, and split into appropriate number of wells in a 96-well round bottom plate for the surface and intracellular cytokine immunostaining. Appropriate isotype control antibodies staining was included as negative controls for the flow cytometry. The panels of antibodies and their corresponding isotype controls used in flow cytometry were described (**Figures 1A–C**; **Supplementary Tables 1**–**3**). Cells were transferred to a 96-well round bottom plate, washed twice in 200 µl of PBS/well, and subjected to surface staining using fluorochrome conjugated monoclonal antibodies (mAbs) against indicated markers and their corresponding isotype controls at previously titrated and optimized dilutions in 50 µl total volume for 30 min at 4°C. Cells were fixed using 1% paraformaldehyde at 4°C for 30 min and resuspended in FACS buffer. Cells were washed once with PBS and permeabilized with 10% Saponin for 45 min at room temperature. Subsequently, cells were washed with Saponin wash (0.1% saponin) and incubated with fluorochrome conjugated mAb against indicated markers and their corresponding isotype controls at previously titrated and optimized dilutions in 50 µl final volume for 45 min at 4°C. Cells were washed once in Saponin wash and stained with indicated secondary antibodies for 45 min at 4°C. Cells were washed once and resuspended in 200 µl FACS buffer and transferred to FACS tubes and acquired using a lymphocyte gate by using BD FACS Aria II machine. For each sample, 100,000 events were acquired. The data were analyzed using FlowJo software (FlowJo V10, Becton, Dickinson& Company; BD) and plotted using GraphPad Prism (GraphPad Prism 5, CA).

### Enzyme-Linked Immunosorbent Assay

IAV specific isotype IgG and SIgA antibodies titers were analyzed by enzyme-linked immunosorbent assay (ELISA) as described previously (30, 31). Briefly, 96-well flat bottom high binding affinity plates were coated with pre-titrated (10 μg/mL) killed virus antigens of IAV H1N1-OH7, H1N2-OH10, or H3N2-OH4 and incubated overnight at 4°C. Plates were washed with PBS-Tween20 (0.05%) and blocked for 2 h with 5% dry milk in PBS-Tween20. Test samples were serially diluted in 2.5% dry milk powder in PBS-Tween20 at a starting dilution of 1:2 for nasal swab and 1:100 for serum, BAL, and lung lysate samples, and 50 μl/well were added to plates and incubated overnight at 4°C. Plates were washed and incubated at RT for 2 h with 50 μl/well of goat anti-pig IgA conjugated with HRP (Bethyl Laboratories Inc., TX) at pre-titrated 1:2,000 dilution or peroxidase labeled AffiniPure Goat Anti-Swine IgG (H+L) (Jackson ImmunoResearch Laboratories Inc., PA) 1:8,000 dilution in 2.5% dry milk in PBS-Tween20. After washing plates were added with a 1:1 mixture of peroxidase substrate solution B and TMB (KPL, MD) (50 μl/well) and incubated for 20 min. The reaction was stopped by adding 1 M phosphoric acid (50 μl/well), and the optical density (OD) was measured by Spectramax microplate reader at 450 nm. The corrected OD values were obtained by subtracting the average value of blank from the test samples.

### Virus Titration

Virus titers were determined as previously described (30, 31). Briefly, 96-well tissue culture plates were seeded with 100 μl/well of MDCK cells (2 × 10^4^ cells/well) in DMEM enriched media and incubated in a 37°C humidified 5% CO_2_ incubator overnight. In another set of sterile 96-well round-bottom plates, a ten-fold serial dilution of nasal swab, BAL fluid, and lung lysate samples were prepared using serum-free DMEM. Plates containing confluent monolayer of MDCK cells were washed three times with sterile 1X PBS and inoculated with 100 μl/well of serially diluted test samples for 1.5 h at 37°C in a 5% CO_2_ incubator followed by 100 μl/well of DMEM serum-free medium containing 2 µg/ml of TPCK-trypsin (Sigma, St. Louis, USA) were added. Plates were incubated for 36 h and fixed with 80% acetone (10 min) for immunostaining. Cells were incubated with (50 μl/well) an Influenza A virus (IAV) nucleoprotein specific mAb (#M058, CalBioreagents, CA) diluted 1:5,000 for 2 h at incubator. Cells were washed and incubated for 1.5 h with (50 μl/well) the secondary antibody Alexa Fluor 488 conjugated goat anti-mouse IgG (H+L) antibody (Life Technologies, OR) at incubator. Mounting media (Glycerol: PBS, pH = 8 at 6:4 proportion) 50 μl/well was added to the immunostained cell plates. Infection was recorded using an Olympus, NY fluorescent microscope, and infectious titer was determined by Reed and Muench method.

### Determination and Scoring of Gross Lung Lesions

Gross lung lesions were assigned a score on the basis of purple-red consolidation of virus affected lesions in each lung lobe. The final lung lesions score was determined by calculating the average of dorsal and ventral lung lobe scores as described previously (32).

### Hemagglutination Inhibition Assay

The virus specific HI titers were determined as described previously (30, 31). Briefly, heat-inactivated test serum samples were 2-fold serially diluted in sterile 1X PBS and incubated for 30 min with 8 HAU (Hemagglutination Units) (25 μl/ml) of H1N2-OH10 or H1N1-OH7 SwIAV. The serum/virus mixture was incubated for 30 min with 1% turkey red blood cells. HI titer in serum is the reciprocal of the last well with complete hemagglutination inhibition.

### Statistical Analysis

Statistical analysis of immune cells data was carried out by using one-way ANOVA followed by Tukey’s post-test. Analysis of titers of IgG and SIgA antibody responses were carried out using two-way ANOVA followed by the Bonferroni test. Data represent the mean value for four to six pigs ± SEM. A *p* < 0.05 was considered statistically significant.

## Results

### Both Nano-11-KAg+Poly(I:C) and Commercial Vaccine Reduced the Challenge Virus Load in the Airways of MDA Positive Pigs and Exhibited Comparable Macroscopic Lung Lesions Scores at DPC6

**Figure 2:**
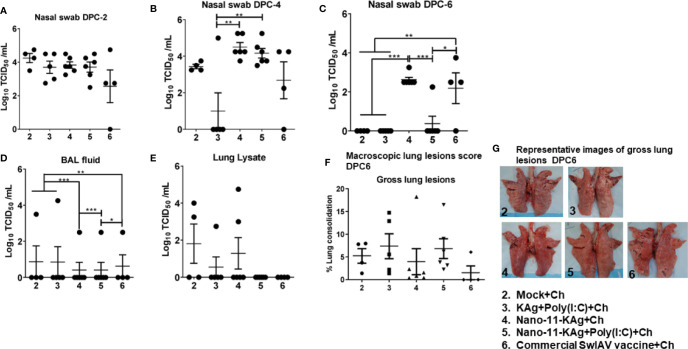
Both Nano-11-KAg+Poly(I:C) and commercial vaccines significantly reduced the challenge virus burden in the nasal passages of MDA-positive pigs and exhibited comparable gross lung pathology scores. Three-week-old maternal antibody positive pigs were administered twice with Nano-11-KAg+Poly(I:C) or as controls mock saline, KAg+Poly(I:C), Nano-11-KAg, or a commercial multivalent SwIAV vaccine and challenged at day post-prime vaccination 35 with H1N1-OH07 SwIAV. Replicating SwIAV H1N1 titers in the respiratory tract was determined by cell culture technique in nasal swab samples at **(A)** DPC2, **(B)** DPC4 and **(C)** DPC6. Also, the infectious virus titers were determined in **(D)** bronchoalveolar lavage (BAL) fluid and **(E)** Lung lysate samples at DPC6. **(F)** The macroscopic lung lesions score was determined at DPC6. **(G)** The representative images of gross lung lesions at DPC6 were displayed. Data represent the mean value of 4–6 pigs ± SEM. Statistical analysis was performed using one-way ANOVA followed by Tukey’s post-test. Asterisk refers to significant difference between the indicated groups (*p < 0.05, **p < 0.01, and *** p < 0.001). Ch: challenge.

To assess the protective efficacy of Nano-11-KAg+Poly(I:C), we analyzed the load of challenge virus (H1N1 SwIAV) in the nasal swab, BAL fluid, and lung lysate samples of piglets, which had high serum titers of SwIAV specific IgG MDA born to vaccinated mothers (**Supplementary Figure 1**). At DPC2 and DPC4, the challenge virus titers were comparable in all the vaccinates except in KAg+Poly(I:C) group at DPC4 (**Figures 2A, B**). A significantly reduced load of challenge virus titers in nasal swab was observed in Nano-11-KAg+Poly(I:C) vaccinates compared to their counterparts from commercial vaccine (*p* < 0.05) and Nano-11-KAg (*p* < 0.001) groups at DPC6 (**Figure 2C**). In addition, both mock challenge and KAg+Poly(I:C) vaccinates also exhibited a significant reduction in the challenge virus titers compared to Nano-11-KAg vaccinates at DPC6 (**Figure 2C**). Challenge virus titers were comparable in all the vaccine groups in BAL fluid at DPC6 (**Figure 2D**). In the lung lysates, the challenge virus titers were undetectable in both Nano-11-KAg+Poly(I:C) and commercial vaccinates at DPC6 (**Figure 2E**). Hence, even in the presence of comparable titers of the challenge virus in the BAL fluid and lung lysate, the Nano-11-KAg+Poly(I:C) vaccinates cleared the challenge virus from nasal passages earlier than their counterparts from commercial SwIAV vaccinates (**Figures 2C–E**). Furthermore, we determined the macroscopic lung lesions scores at DPC6. Although statistically not significant, both Nano-11-KAg [5 out of 6 animals] and Nano-11-KAg+Poly(I:C) [5 out of 6 animals] vaccinates exhibited comparable gross lesions scores of lungs to their counterparts from commercial vaccine group (**Figures 2F, G**). These findings confirm that the monovalent Nano-11-KAg+Poly(I:C) vaccine offered protection in terms of reduced virus load and lung pathology comparable to the trivalent commercial SwIAV vaccine.

### Phenotypes of CTLs (CD3^+^CD4^-^CD8α^+^β^+^) in TBLN: H1N2-SwIAV Specific CTLs in Nano-11-KAg+Poly(I:C) Vaccinates and in Commercial Vaccinates CTLs With IL-17A^+^ and Early Effector and H1N1-Specific CTLs With IL-17A^+^, IFNγ^+^&TNFα^+^, and Early Effector Phenotypes Were Elicited at DPC6 in MDA-Positive Pigs

Since the Nano-11-based vaccine was delivered intranasal, to elucidate the underlying mechanisms of protection, cell-mediated immune responses in TBLN MNCs at DPC6 were characterized. We characterized CTLs for the expression of Perforin and CD27 to determine the differentiation stage of the cells, and their cytokine expression patterns. The co-stimulatory molecule CD27 is a member of the tumor necrosis factor receptor (TNFR) family. CD27/CD70 interaction leads to the expansion of effector cells, along with enhanced survivability and differentiation of memory T-cells. Based on age-related changes, Perforin^+^ CD8 T-cells can be differentiated into early effector [Perforin^+^CD27^+^] and late effector [Perforin^+^CD27^-^] CTLs (25).

**Figure 3:**
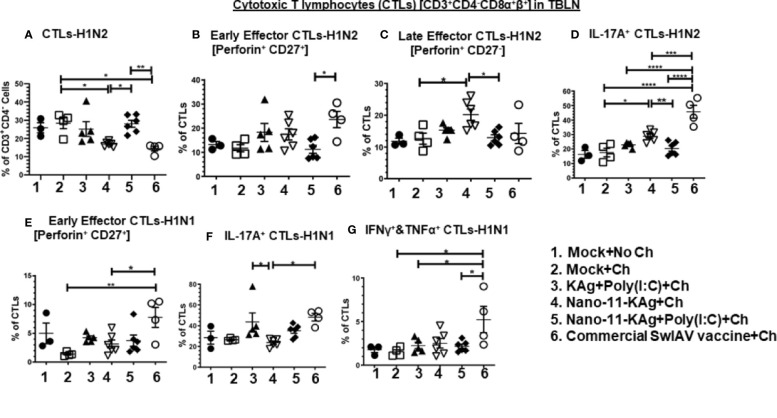
Nano-11-KAg+Poly(I:C) vaccine triggered H1N2- and H1N1-specific CTL responses in tracheobronchial lymph nodes (TBLN) of MDA-positive pigs. Three-week-old maternal antibody positive pigs were administered twice with Nano-11-KAg+Poly(I:C) or controls mock saline, KAg+Poly(I:C), Nano-11-KAg or commercial multivalent SwIAV vaccine and challenged at day post prime vaccination 35 with H1N1-OH07 SwIAV. TBLN MNCs isolated at DPC6 were stimulated with H1N2-OH10 and H1N1-OH7 SwIAV for 72 h *in vitro*. The cells were immunostained and analyzed by flow cytometry for the frequencies of different types of CTLs [CD3^+^CD4^-^CD8α^+^β^+^] specific to **(A)** H1N2; Early effector CTLs [Perforin^+^CD27^+^] specific to **(B)** H1N2 and **(E)** H1N1; Late effector CTLs [Perforin^+^CD27^-^] specific to **(C)** H1N2; IL-17A^+^ CTLs specific to **(D)** H1N2 and **(F)** H1N1; IFNγ^+^&TNFα^+^ CTLs specific to **(G)** H1N1. Data represent the mean value for 4 to 6 pigs ± SEM. Statistical analyses were carried out by One-way ANOVA with Tukey’s post-test: *p < 0.05, **p < 0.01, ***p < 0.001, and ****p < 0.0001. Ch: challenge.

The frequency of H1N2-specific CTLs was significantly increased in Nano-11-KAg+Poly(I:C) vaccinates (*p* < 0.01) compared to their counterparts from the commercial vaccine and Nano-11-KAg groups (*p* < 0.05) (**Figure 3A**). There was a significant increase in the proportion of H1N2- and H1N1-specific early effector CTLs in commercial vaccinates compared to Nano-11-KAg+Poly(I:C) (*p* < 0.05), Nano-11-KAg (*p* < 0.05) and mock challenge (*p* < 0.01) groups, respectively (**Figures 3B, E**). However, a significant upregulation of H1N2-specific late effector CTLs frequency was observed in Nano-11-KAg vaccinates compared to mock challenge (*p* < 0.05) and Nano-11-KAg+Poly(I:C) (*p* < 0.05) groups (**Figure 3C**). The commercial vaccinates displayed significantly higher frequencies of H1N2-specific IL-17A^+^ CTLs compared to all other groups (*p* < 0.05 to *p* < 0.0001), and H1N1-specific IL-17A^+^ CTLs compared to the Nano-11-KAg group (*p* < 0.05) (**Figures 3D, F**). KAg+Poly(I:C) vaccinates also upregulated H1N1-specific IL-17A^+^ CTLs frequencies compared to Nano-11-KAg group (*p* < 0.05) (**Figure 3F**). In Nano-11-KAg vaccinates a significant upregulation of H1N2-specific IL-17A^+^ CTLs compared to Nano-11-KAg+Poly(I:C) (*p* < 0.01) and mock challenge (*p* < 0.05) groups was observed (**Figure 3D**). The commercial vaccinates exhibited significantly higher frequencies of H1N1-specific IFNγ^+^&TNFα^+^ CTLs compared to Nano-11-KAg+Poly(I:C), KAg+Poly(I:C), and mock challenge groups (*p* < 0.05) (**Figure 3G**). Overall, these results demonstrated that Nano-11-KAg+Poly(I:C) vaccine elicited a strong H1N2-specific CTLs response, while in commercial vaccinates, IL-17A^+^ CTLs, and early effectors, and H1N1-specific IL-17A^+^, IFNγ^+^&TNFα^+^ CTLs and early effector phenotypes were observed in MDA-positive pigs at DPC6.

### Phenotypes of T-Helper/Memory Cells (CD3^+^CD4^+^CD8α^+^β^-^) in TBLN: Commercial Vaccine Stimulated H1N2- and H1N1-Specific IFNγ^+^&TNFα^+^, Central Memory and IL-17A^+^, and Nano-11-KAg+Poly(I:C) Elicited H1N2-Specific Central Memory, IFNγ^+^ and IFNγ^+^&TNFα^+^ and H1N1-Specific IL-17A^+^ T-Helper/Memory Cells at DPC6 in MDA-Positive Pigs

Based on the expression of CD27, T-helper/Memory cells can be differentiated into central memory [CD27^+^] and effector memory [CD27^-^] cells. Whereas central memory T-helper/Memory cells exhibit high proliferation potential and intermediate cytokine secretion profile, the effector memory cells are characterized by the lowest proliferation and highest ability to produce IFNγ and TNFα (33). We also analyzed the functional ability of antigen-specific T-helper/Memory cells to generate different cytokines.

**Figure 4:**
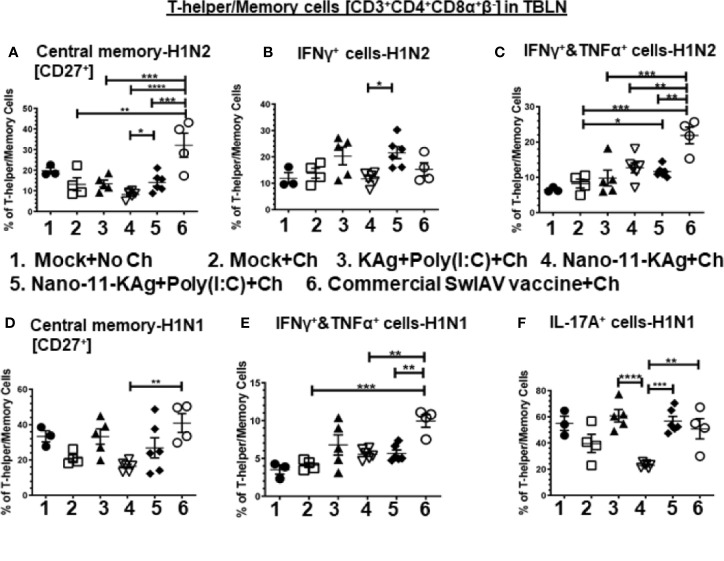
Nano-11-KAg+Poly(I:C) vaccine stimulated H1N2- and H1N1-specific T-helper/Memory cell responses in TBLN of MDA-positive pigs. Three-week-old maternal antibody positive pigs were administered twice with Nano-11-KAg+Poly(I:C) or controls mock saline, KAg+Poly(I:C), Nano-11-KAg or commercial multivalent SwIAV vaccine and challenged at day post-prime vaccination 35 with H1N1-OH07 SwIAV. TBLN MNCs isolated at DPC6 were stimulated with H1N2-OH10 and H1N1-OH7 SwIAV for 72 h *in vitro*. The cells were immunostained and analyzed by flow cytometry for frequencies of different types of T-helper/Memory cells [CD3^+^CD4^+^CD8α^+^β^-^] - central memory cells [CD27^+^] specific to **(A)** H1N2 and **(D)** H1N1; IFNγ^+^ cells specific to **(B)** H1N2; IFNΥ^+^&TNFα^+^ cells specific to **(C)** H1N2 and **(E)** H1N1; and IL-17A^+^ cells specific to **(F)** H1N1. Data represent the mean value for 4 to 6 pigs ± SEM. Statistical analyses were carried out by One-way ANOVA with Tukey’s post-test - * (*p <* 0.05), ** (*p <* 0.01), *** (*p <* 0.001), ****(*p <* 0.0001). Ch: challenge.

Nano-11-KAg+Poly(I:C) vaccinates displayed significantly higher H1N2-specific central memory T-helper/Memory cell frequencies compared to Nano-11-KAg (*p* < 0.05) (**Figure 4A**). While commercial vaccine elicited a significantly higher H1N2-specific central memory cells compared to all other groups (*p* < 0.01 to *p* < 0.0001), and H1N1-specific central memory cells compared to Nano-11-KAg (*p* < 0.01) group (**Figures 4A, D**). Remarkably, in Nano-11-KAg+Poly(I:C) vaccinates, a significant upregulation of H1N2-specific IFNγ^+^ T-helper/Memory cell frequencies compared to Nano-11-KAg group (*p* < 0.05) and IFNγ^+^&TNFα^+^ cells compared to mock challenge (*p* < 0.05) were detected (**Figures 4B, C**). However, commercial vaccinates exhibited a significantly higher H1N2- and H1N1-specific IFNγ^+^&TNFα^+^ T-helper/Memory cell frequencies compared to all other vaccinates [*p* < 0.01 to *p* < 0.001] (**Figures 4C, E**). Both Nano-11-KAg+Poly(I:C) (*p* < 0.001) and commercial vaccines (*p* < 0.01) induced significantly higher H1N1-specific IL-17A^+^ T-helper/Memory cell frequencies compared to Nano-11-KAg group [*p* < 0.01 to *p* < 0.001] (**Figure 4F**). In addition, KAg+Poly(I:C) vaccinates displayed significantly higher H1N1-specific IL-17A^+^ T-helper/Memory cell frequencies (*p* < 0.0001) compared to Nano-11-KAg group (**Figure 4F**). Taken together, these data suggested that Nano-11-KAg+Poly(I:C) vaccinates exhibited potent H1N2- and H1N1-specific polyfunctional T-helper/Memory cell responses comparable to the trivalent commercial vaccine in TBLN of MDA-positive pigs at DPC6.

### Phenotypes of H1N2-Specific CTLs and T-Helper/Memory Cells in Blood: Nano-11-KAg+Poly(I:C) Vaccine Boosted IFNγ^+^ CTLs and the Commercial Vaccine Boosted IL-17A^+^, IFNγ^+^ and Early Effector CTLs, and Central Memory, IFNγ^+^&TNFα^+^ T-Helper/Memory Cell Responses in MDA-Positive Pigs

**Figure 5:**
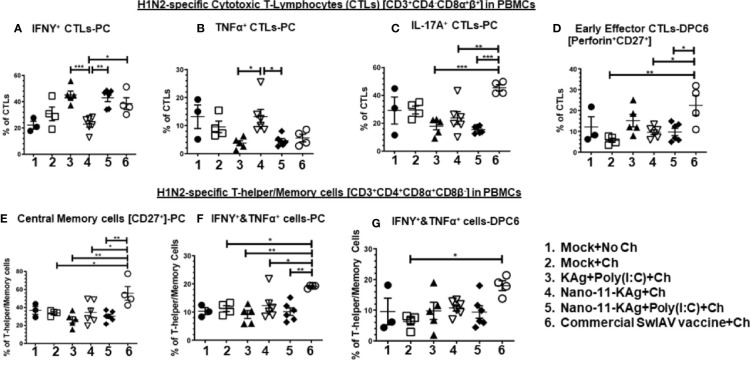
Nano-11-KAg+Poly(I:C) vaccine induced systemic H1N2-specific CTLs and T-helper/Memory cell responses in MDA-positive pigs. Three-week-old maternal antibody positive pigs were administered twice with Nano-11-KAg+Poly(I:C) or controls mock saline, KAg+Poly(I:C), Nano-11-KAg or commercial multivalent SwIAV vaccine and challenged at day post-prime vaccination 35 with H1N1-OH07 SwIAV. PBMCs isolated at day post-prime vaccination 34 (Prechallenge; PC) and at DPC6 were stimulated with H1N2-OH10 SwIAV for 72 h *in vitro*. The cells were immunostained and analyzed by flow cytometry for frequencies of different types of CTLs [CD3^+^CD4^_^CD8α^+^β^+^]: IFNγ^+^ CTLs at **(A)** PC; TNFα^+^ CTLs at **(B)** PC; IL-17A^+^ CTLs at **(C)** PC; Early effector CTLs [Perforin^+^CD27^+^] at **(D)** DPC6; and T-helper/Memory cells [CD3^+^CD4^+^CD8α^+^8β^-^]: Central memory cells at **(E)** PC; IFNΥ^+^&TNFα^+^ cells at **(F)** PC and at **(G)** DPC6. Data represent the mean value of 4 to 6 pigs ± SEM. Statistical analyses were carried out by One-way ANOVA with Tukey’s post-test - * (*p <* 0.05), ** (*p <* 0.01), *** (*p <* 0.001). Ch: Challenge.

PBMCs isolated at day 34 post vaccination (prechallenge, PC) and DPC6 were analyzed for phenotypes of activated CTLs and T-helper/Memory cells. Our data indicated a significant enhancement of IFNγ^+^ CTLs in Nano-11-KAg+Poly(I:C) (*p* < 0.01), commercial vaccine (*p* < 0.05) and KAg+Poly(I:C) (*p* < 0.001) groups compared to Nano-11+KAg vaccinates at PC (**Figure 5A**). In Nano-11+KAg vaccinates, TNFα^+^ CTLs frequencies were upregulated compared to KAg+Poly(I:C) (*p* < 0.05) and Nano-11-KAg+Poly(I:C) (*p* < 0.05) at PC (**Figure 5B**). The commercial vaccine administered animals had significantly higher IL-17A^+^ CTLs compared to all other groups (*p* < 0.01 to *p* < 0.001) at PC (**Figure 5C**). The frequency of early effector CTLs at DPC6 was significantly increased in the commercial vaccine group compared to all other groups (*p* < 0.05 to *p* < 0.01) at DPC6 (**Figure 5D**). Overall, systemically Nano-11-KAg+Poly(I:C) vaccine elicited H1N2-specific IFNγ^+^ CTLs at PC and commercial vaccine stimulated IFNγ^+^, IL-17A^+^ CTLs at PC and early effector CTLs at DPC6. Furthermore, commercial SwIAV vaccine stimulated significantly higher H1N2-specific central memory cells (*p* < 0.05 to *p* < 0.01) compared to all other groups at PC (**Figure 5E**), IFNγ^+^&TNFα^+^ T-helper/Memory cells compared to all other groups (*p* < 0.05 to *p* < 0.01) at PC (**Figure 5F**), and also at DPC6 compared to mock challenge (*p* < 0.05) (**Figure 5G**).

### Phenotypes of H1N1-Specific CTLs and T-Helper/Memory Cells in Blood: Nano-11-KAg+Poly(I:C) Invoked IFNγ^+^ T-Helper/Memory Cells While Commercial Vaccine Augmented IFNγ^+^&TNFα^+^ CTLs and IL-17A^+^, IFNγ^+^&TNFα^+^ and Central Memory T-Helper/Memory Cells in MDA-Positive Pigs

**Figure 6:**
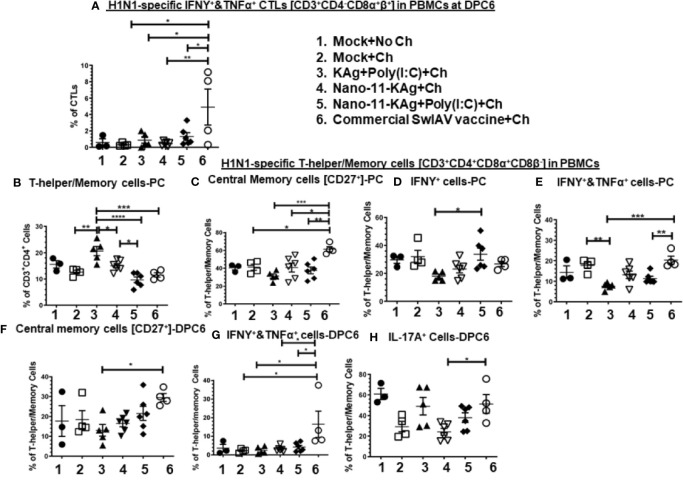
Nano-11-KAg+Poly(I:C) vaccine generated systemic H1N1-specific CTLs and T-helper/Memory cell responses in MDA-positive pigs. Three-week-old maternal antibody positive pigs were administered twice with Nano-11-KAg+Poly(I:C) or controls mock saline, KAg+Poly(I:C), Nano-11-KAg or commercial multivalent SwIAV vaccine and challenged at day post-prime vaccination 35 with H1N1-OH07 SwIAV. PBMCs isolated at day post-prime vaccination 34 (Prechallenge; PC) and DPC6 were stimulated with H1N1-OH7 SwIAV KAg at 10 µg/ml for 72 h *in vitro*. The cells were immunostained and analyzed by flow cytometry for frequencies of H1N1 specific different types of CTLs [CD3^+^CD4^_^CD8α^+^β^+^]: IFNΥ^+^&TNFα^+^ cells at **(A)** DPC6; T-helper/Memory cells [CD3^+^CD4^+^CD8α^+^8β^-^] at **(B)** PC; Central memory cells [CD27^+^] at **(C)** PC and **(F)** DPC6; IFNγ^+^ cells at **(D)** PC; IFNΥ^+^&TNFα^+^ cells at **(E)** PC and **(G)** DPC6; IL-17A^+^ cells at **(H)** DPC6. Data represent the mean value for 4 to 6 pigs ± SEM. Statistical analyses were carried out by One-way ANOVA with Tukey’s post-test - * (*p <* 0.05), ** (*p <* 0.01), *** (*p <* 0.001), ****(*p <* 0.0001). Ch: challenge.

The commercial vaccine administered animals had significantly upregulated (*p* < 0.05 to *p* < 0.01) IFNγ^+^&TNFα^+^ CTLs frequencies compared to all other groups in PBMCs at DPC6 (**Figure 6A**).

Nano-11-KAg vaccinates displayed a significant upregulation of T-helper/Memory cell frequencies compared to Nano-11-KAg+Poly(I:C) and KAg+Poly(I:C) vaccinates (*p* < 0.05) at PC (**Figure 6B**). Likewise, KAg+Poly(I:C) vaccinates also had significantly higher T-helper/Memory cells compared to all other groups at PC (*p* < 0.05 to *p* < 0.0001) (**Figure 6B**). Remarkably, Nano-11-KAg+Poly(I:C) vaccinates displayed significantly higher (*p* < 0.05) IFNγ^+^ T-helper/Memory cells frequencies compared to KAg+Poly(I:C) group animals at PC (**Figure 6D**). However, the commercial vaccine group animals had significantly upregulated central memory T-helper/Memory cell frequencies compared to all other groups (*p* < 0.05 to *p* < 0.001) at PC (**Figure 6C**) and also at DPC6 compared to KAg+Poly(I:C) group (*p* < 0.05) (**Figure 6F**). The commercial vaccine administered animals also had upregulated IFNγ^+^&TNFα^+^ T-helper/Memory cell frequencies compared to Nano-11-KAg+Poly(I:C) (*p* < 0.01) and KAg+Poly(I:C) (*p* < 0.001) at PC (**Figure 6E**), and at DPC6 compared to all other groups (*p* < 0.05) (**Figure 5G**). Furthermore, the commercial vaccinates had significantly higher IL-17A^+^ T-helper/Memory cell frequencies compared to Nano-11-KAg group (*p* < 0.05) at DPC6 (**Figure 6H**). Overall, the commercial vaccine elicited H1N1-specific enhanced IFNγ^+^&TNFα^+^ CTLs and T-helper/Memory cells with IL-17A^+^, IFNγ^+^&TNFα^+^, and central memory phenotypes, while Nano-11-KAg+Poly(I:C) vaccine stimulated a strong heterologous IFNγ^+^ T-helper/Memory cell response systemically in MDA-positive pigs.

### T-Helper [CD3^+^CD4^+^CD8α^-^β^-^CD27^-^] and Naïve T-Helper Cell [CD3^+^CD4^+^CD8α^-^β^-^CD27^+^] Populations: Commercial Vaccine Induced Excess of H1N1-Specific Both These Two Cell Types in TBLN, and H1N2-Specific Only Naïve T-Cells in Blood, While Only H1N2- and H1N1-Specific T-Helper Cells Were Augmented in Nano-11-KAg+Poly(I:C) Vaccinates at Both Systemic and Mucosal Sites in MDA-Positive Pigs

We further characterized the T-helper cell phenotypes by recall assays *in vitro*. Porcine naïve T-helper cells express the co-stimulatory molecule CD27 [CD3^+^CD4^+^CD8α^-^β^-^CD27^+^]. Upon restimulation, the memory cells undergo secondary expansion and give rise to different phenotypes (33).

**Figure 7:**
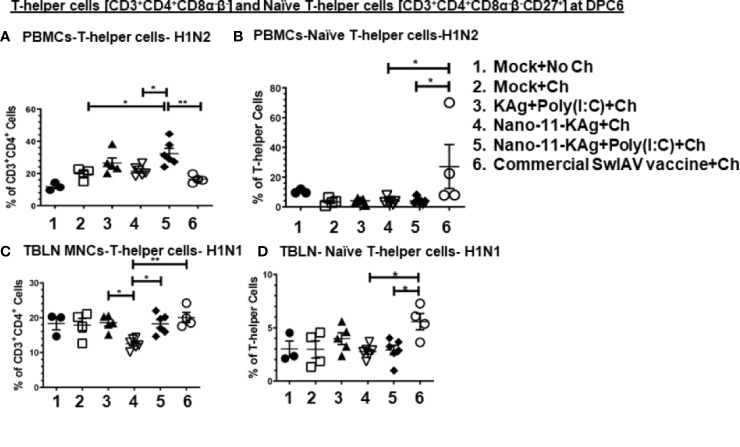
Commercial vaccine resulted in the accumulation of naïve T-helper cells in both peripheral blood and the TBLN of MDA-positive pigs. Three-week-old maternal antibody positive pigs were administered twice with Nano-11-KAg+Poly(I:C) or controls mock saline, KAg+Poly(I:C), Nano-11-KAg or commercial multivalent SwIAV vaccine and challenged at day post-prime vaccination 35 with H1N1-OH07 SwIAV. PBMCs isolated at day post-prime vaccination 34 (Prechallenge; PC) and DPC6 and TBLN MNCs isolated at DPC6 were stimulated with H1N2-OH10 and H1N1-OH7 SwIAV for 72 h *in vitro*. The cells were immunostained and analyzed by flow cytometry for frequencies of total T-helper cells [CD3^+^CD4^+^CD8α^-^β^-^] and naïve T-helper cells [CD3^+^CD4^+^CD8α^-^β^-^CD27^+^]. T-helper cells specific to H1N2 at **(A)** DPC6; Naïve T-helper cells specific to H1N2 at **(B)** DPC6; T-helper cells in TBLN MNCs specific to H1N1 at **(C)** DPC6; and Naïve T-helper cells specific to H1N1 at **(D)** DPC6. Data represent the mean value for 4 to 6 pigs ± SEM. Statistical analyses were carried out by One-way ANOVA with Tukey’s post-test- * (*p <* 0.05), ** (*p <* 0.01), *** (*p <* 0.001), ****(*p <* 0.0001). Ch: challenge.

Nano-11-KAg+Poly(I:C) vaccinates displayed significantly upregulated frequencies of H1N2-specific T-helper cells compared to the commercial vaccine (*p* < 0.01), Nano-11-KAg and mock challenge groups (*p* < 0.05) in PBMCs at DPC6 (**Figure 7A**). While commercial vaccine administered animals exhibited significantly increased frequencies of H1N2-specific naïve T-helper cell (CD3^+^CD4^+^CD8α^-^β^-^CD27^+^) frequencies compared to Nano-11-KAg+Poly(I:C) and Nano-11-KAg (*p* < 0.05) in PBMCs at DPC6 (**Figure 7B**). A similar trend was observed in the lung draining TBLN at DPC6 (**Figure 7C**). Both commercial vaccine (*p* < 0.01) and Nano-11-KAg+Poly(I:C) vaccinates (*p* < 0.05) upregulated H1N1-specific T-helper cell frequencies compared to Nano-11-KAg group at DPC6 (**Figure 7C**). The KAg+Poly(I:C) vaccinates also displayed a significantly higher H1N1-specific T-helper cells compared to the Nano-11-KAg group (*p* < 0.05) (**Figure 7C**). Furthermore, the commercial vaccine group animals exhibited significantly higher H1N1-specific naïve T-helper cell frequencies compared to both Nano-11-KAg and Nano-11-KAg+Poly(I:C) groups (*p* < 0.05) (**Figure 7D**) in TBLN MNCs at DPC6. These results indicated that the administration of commercial vaccine resulted in likely inadequate antigen processing/presentation leading to inefficient differentiation of T-helper cells resulting in the accumulation of naïve T-helper cells in both TBLN and blood of MDA-positive pigs at DPC6. Since naïve T-helper cell (CD3^+^CD4^+^CD8α^-^β^-^CD27^+^) mentioned above responded to restimulation *ex vivo* it may not be appropriate to list them as naïve cells based on the phenotype alone, but in earlier study, similar restimulated cells were grouped under naïve T-helper cells (33).

### Antibody Responses in Vaccinates: Nano-11-KAg+Poly(I:C) Vaccine Stimulated Robust Cross-Reactive Localized IgG and SIgA Responses in Lungs While the Commercial Vaccine Elicited Significantly Higher Levels of Serum and Lung IgG Titers in MDA Positive Pigs

**Figure 8:**
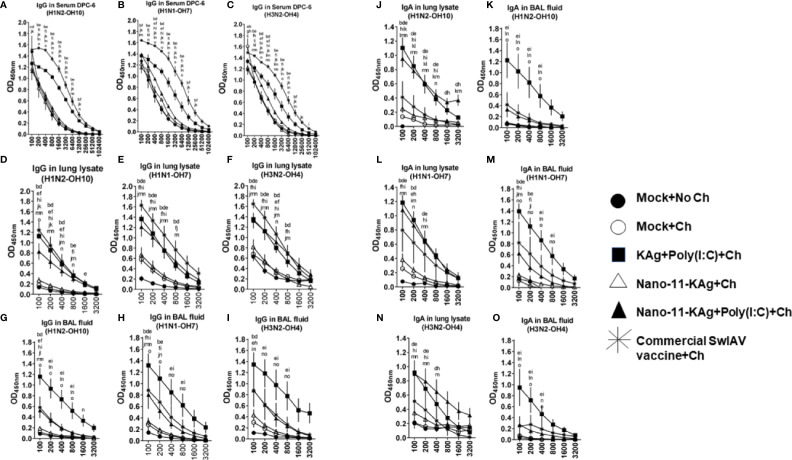
Nano-11-KAg+Poly(I:C) vaccine induced robust localized IgG and SIgA responses in the airways of MDA-positive pigs. Three-week-old maternal antibody positive pigs were administered twice with Nano-11-KAg+Poly(I:C) or controls mock saline, KAg+Poly(I:C), Nano-11-KAg or commercial multivalent SwIAV vaccine and challenged at day post-prime vaccination 35 with H1N1-OH07 SwIAV. Samples collected at DPC6 were used for IgG antibody analysis in serum, lung lysate, and BAL fluid against **(A, D, G)** H1N2-OH10; **(B, E, H)** H1N1-OH7; and **(C, F, I)** H3N2-OH4 SwIAVs by ELISA. Data represent the mean value of 4 to 6 pigs ± SEM. The SIgA antibody response in lung lysate and BAL fluid samples against **(J, K)** H1N2-OH10; **(L, M)** H1N1-OH7; and **(N, O)** H3N2-OH4 SwIAVs were analyzed by ELISA. Statistical analysis was carried out using two-way ANOVA followed by the Bonferroni test. Each letter indicates the significant difference between the groups at the indicated dilution. The letters a, b, c, d, and e indicate the difference between the mock group compared to Mock+Ch, Commercial SwIAV vaccine+Ch, Nano-11-KAg+Ch, Nano-11-KAg+Poly(I:C)+Ch, and KAg+Poly(I:C)+Ch, respectively. The letters f, g, h, and i indicate the difference between Mock+Ch group compared to Commercial SwIAV vaccine+Ch, Nano-11-KAg+Ch, Nano-11-KAg+Poly(I:C)+Ch and KAg+Poly(I:C)+Ch, respectively. The letters j, k, and l indicate the difference between Commercial SwIAV vaccine+Ch compared to Nano-11-KAg+Ch, Nano-11-KAg+Poly(I:C)+Ch and KAg+Poly(I:C)+Ch, respectively. The letter m indicates the difference between Nano-11-KAg+Ch and Nano-11-KAg+Poly(I:C)+Ch. The letter n and o indicates the difference between KAg+Poly(I:C)+Ch group compared to Nano-11-KAg+Ch and Nano-11-KAg+Poly(I:C)+Ch groups, respectively.

We characterized SwIAV specific humoral immune responses to understand their contribution towards protection. We investigated the H1N2-, H1N1- and heterosubtypic H3N2 IAV-specific antibody responses both at systemic and mucosal compartments of MDA-positive pigs. The commercial vaccine received animals exhibited significantly higher IgG titers in serum (**Figures 8A–C**) and lung lysate samples compared to Nano-11 based vaccine groups at DPC6 (**Figures 8D–F**). In contrast, in the BAL fluid, a significantly higher H1N2-, H1N1- and H3N2- SwIAV-specific IgG titers were displayed by KAg+Poly(I:C), Nano-11-KAg+Poly(I:C), and commercial vaccine group animals compared to Nano-11-KAg and mock challenge groups at DPC6 (**Figures 8G–I**). The Nano-11-KAg+Poly(I:C) and KAg+Poly(I:C) vaccinates also elicited robust H1N2-, H1N1- and H3N2- IAV-specific SIgA responses in lung lysate (**Figures 8J, L, N**) compared to the commercial vaccine and other control groups at DPC6. In addition, KAg+Poly(I:C) vaccinates displayed significantly higher SIgA titers in the BAL fluid compared to other groups (**Figures 8K, M, O**). These results suggested that both Nano-11-KAg+Poly(I:C) and KAg+Poly(I:C) vaccines elicited robust homologous, heterologous and heterosubtypic IAV-specific SIgA and IgG responses in the lungs, while the commercial vaccine induced significant levels of serum and lung IgG responses in MDA-positive pigs. It is surprising that in commercial vaccinates also robust antibody responses were observed even in the presence of MDA which needs further investigation.

### Commercial Vaccine Elicited Strong Systemic HI Antibody Response in MDA Positive Pigs

**Figure 9:**
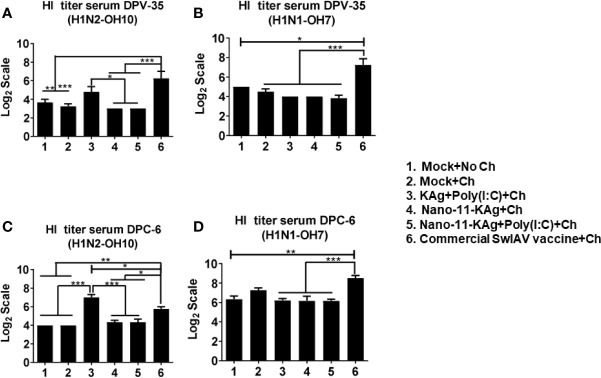
Commercial vaccine elicited strong systemic HI antibody response in MDA-positive pigs. Three-week-old maternal antibody positive pigs were administered twice with Nano-11-KAg+Poly(I:C) or controls mock saline, KAg+Poly(I:C), Nano-11-KAg or commercial multivalent SwIAV vaccine and challenged at day post-prime vaccination 35 with H1N1-OH07 SwIAV. Serum HI antibody titers were measured at: **(A, B)** day post-vaccination (DPV) 35 and **(C, D)** DPC 6 against **(A, C)** H1N2-OH10 and **(B, D)** H1N1-OH7 SwIAVs. Data represent the mean value of 4 to 6 pigs ± SEM. Statistical analysis was performed using one-way ANOVA followed by Tukey’s post-test. Asterisk refers to statistical difference between the indicated groups (*p < 0.05, **p < 0.01, and *** p < 0.001).

Consistent with the augmented systemic IgG response in commercial SwIAV vaccinates, a significantly higher H1N2- and H1N1- SwIAV-specific serum HI antibody titers at both DPV35 and DPC6 compared to their counterparts from Nano-11 based SwIAV vaccine groups was observed (**Figures 9A–D**). An increase in H1N2-specific HI titers was also detected in KAg+Poly(I:C) vaccinates at DPV35 and DPC6 respectively (**Figures 9A, C**). It is evident that parenterally administered commercial vaccine induces significantly higher SwIAV-specific serum HI antibody response in MDA-positive pigs.

## Discussion

The holy grail in modern vaccinology is the “Rational Vaccine Design” wherein we strive to improve the vaccine efficacy by adding different components including adjuvants. A systems-wide analysis of the adjuvant Poly(I:C) has been shown to augment induction of diverse innate signaling pathways such as Toll-like receptor-7 (TLR-7), TLR-4, retinoic acid inducible gene-I (RIG-I), melanoma differentiation-associated protein-5 (MDA-5), protein kinase RNA-activated (PKR), type I interferons (IFNs), complement, and inflammasomes identical to that of a live attenuated yellow fever vaccine in humans (9), confirming the potency and authenticity of Poly(I:C) as an efficacious adjuvant. In our study, the monovalent Nano-11 based Poly(I:C) adjuvanted KAg vaccine of SwIAV [Nano-11-KAg+Poly(I:C)] reduced the challenge virus load in the airways of MDA-positive pigs and exhibited comparable macroscopic lung lesions scores to that of the trivalent commercial SwIAV vaccine at DPC6. Mechanistically, Poly(I:C) augmented the antigen-specific cell-mediated and humoral immune responses of intranasally co-delivered monovalent Nano-11-KAg at both mucosal and systemic compartments of MDA-positive pigs. Particularly, Nano-11-KAg+Poly(I:C) enhanced the cross-reactive SwIAV-specific polyfunctional H1N2-specific T-helper/Memory cells with central memory, IFNγ^+^, IFNγ^+^&TNFα^+^, and heterologous H1N1-specific IL-17A^+^ phenotypes; and also enhanced H1N2-specific CTLs frequencies in the TBLN of pigs. While the commercial vaccine upregulated H1N2-specific IL-17A^+^ and early effector CTLs and H1N1-specific IL-17A^+^, IFNγ^+^&TNFα^+^ and early effector CTLs in TBLN of MDA-positive pigs. Furthermore, the commercial vaccine also elicited H1N2- specific IFNγ^+^&TNFα^+^ and central memory T-helper/Memory cells, and H1N1-specific IL-17A^+^, IFNγ^+^&TNFα^+^ and central memory T-helper/Memory cells in TBLN. Importantly, the commercial vaccine did not elicit H1N2-specific IFNγ single positive T-helper/Memory T-cells in the TBLN. In addition, both Nano-11-KAg+Poly(I:C) and commercial SwIAV vaccine stimulated the upregulation of H1N1-specific T-helper cells in TBLN at DPC6. However, only commercial SwIAV vaccinates exhibited robust accumulation of H1N1-specific naïve T-helper cells in the TBLN, suggesting the possible unknown mechanism of cellular responses in MDA-positive pigs, which needs investigation.

In the systemic compartment, Nano-11-KAg+Poly(I:C) vaccinates upregulated H1N2-specific IFNγ^+^ CTLs at prechallenge and displayed downregulation of early effector CTL frequencies at DPC6. Likewise, there was a significant decrease of IFNγ^+^&TNFα^+^and central memory T-helper/Memory cell frequencies at both PC and DPC6. While the commercial vaccine elicited a strong H1N2-specific IFNγ^+^&TNFα^+^ and central memory T-helper/Memory responses at prechallenge and IFNγ^+^&TNFα^+^ T-helper/Memory cells at DPC6; IL-17A^+^ and IFNγ^+^ CTLs at prechallenge and early effector CTLs at DPC6. Like the trend in TBLN, commercial SwIAV vaccinates displayed excess accumulation of H1N2-specific naïve T-helper cells at DPC6. While Nano-11-KAg+Poly(I:C) augmented only H1N2-specific T-helper cell population in blood at DPC6. These data indicated vast differences in the expression of different phenotypes of CTLs, T-helper cells, and T-helper/Memory cells by Nano-11 and Poly(I:C) based SwIAV monovalent vaccine compared to a multivalent commercial vaccine in MDA-positive nursery pigs.

When SwIAV H1N1-specific immune cell profiles were analyzed in the blood, Nano-11-KAg+Poly(I:C) vaccine augmented IFNγ^+^ T-helper/Memory cell responses at prechallenge. While commercial vaccine elicited IFNγ^+^&TNFα^+^ CTLs and IFNγ^+^&TNFα^+^ and central memory T-helper/Memory cells at prechallenge; and IFNγ^+^&TNFα^+^, IL-17A^+^ and central memory T-helper/Memory cells at DPC6. These findings indicated that in commercial vaccine received animals, the kinetics of systemic H1N1-specific cell-mediated immune responses were more robust and of a strong inflammatory nature compared to Nano-11-KAg+Poly(I:C) vaccine induced responses in MDA-positive pigs.

Nano-11-KAg+Poly(I:C) elicited homologous, heterologous, and heterosubtypic SIgA and IgG antibody responses within lungs. This result is consistent with a published literature in mice. Activation of distinct combination of TLRs in dendritic cells leads to synergistic secretion of proinflammatory cytokines leading to the induction of potent, antigen-specific, neutralizing antibodies. Administration of PLGA nanoparticles consisting of TLR-4 ligand monophosphoryl lipid A (MPL) and TLR-7 ligand imiquimod (R837) together with an antigen in separate nanoparticles to mice stimulated potent humoral immune responses (34). In our study in MDA-positive pigs, Poly(I:C) in combination with Nano-11-KAg activated induction of robust antigen-specific both mucosal and systemic antibody and cellular responses, while the commercial SwIAV vaccine stimulated enhanced mainly the systemic IgG and cellular responses.

In the lung draining TBLN, Nano-11-KAg+Poly(I:C) vaccine induced increased frequencies of H1N2-specific total CTLs with enhanced generation of T-helper/Memory cells having central memory phenotypes, and polyfunctional activity (IFNγ^+^, IFNγ^+^&TNFα^+^). Furthermore, it also elicited H1N1-specific IL-17A^+^ T-helper/Memory cell frequencies. These results indicated the induction of a robust and cross-reactive cell-mediated immune response by Nano-11-KAg+Poly(I:C) vaccine in the TBLN. In contrast, in commercial vaccinates, the H1N2-specific response was characterized by augmented early effector CTLs and CTLs expressing IL-17A and central memory phenotypes, and T-helper/Memory cells expressing IFNγ &TNFα. In addition, observed H1N1-specific increased early effector and polyfunctional (IL-17A^+^ and IFNγ^+^&TNFα^+^) CTLs and activated central memory, IFNγ^+^&TNFα^+^, and IL-17A^+^T-helper/Memory cells, with excess accumulation of naive T-helper cell populations. Taken together, these data suggested that the commercial vaccine elicits much slower kinetics and inefficient cell-mediated immune responses compared to Nano-11-KAg+Poly(I:C) vaccine at both the systemic and mucosal compartments in MDA-positive pigs.

In intranasal Nano-11-KAg+Poly(I:C) vaccinates, robust SwIAV-specific cell-mediated immune responses were mediated likely through efficient induction of innate immune pathways involved in antigen recognition/presentation/activation such as apoptosis-associated speck-like protein containing a CARD (ASC)/Caspase-1 inflammasomes, dendritic cell (DC) maturation, nuclear factor-κB (NF-κB) signaling needed for mounting protective cognate adaptive immune responses (9, 12, 35). This leads to enhanced differentiation of naïve T-helper cells into effector T-helper/Memory cells [CD3^+^CD4^+^CD8α^+^CD8β^-^CD27^-^], which are capable of secreting high amounts of effector cytokines leading to efficient clearance of heterologous challenge virus at the site of infection. Unlike the commercial vaccine, the cytokine and cellular signatures in Nano-11-KAg+Poly(I:C) vaccinates were dominated by IFNγ single positive and central memory T-helper/Memory cells and polyfunctional T-helper/Memory cells (IFNγ^+^&TNFα^+^ and IL-17A^+^) leading to induction of strong cell-mediated immune responses. In contrast, the commercial vaccine elicited predominant IL-17A, IFNγ^+^&TNFα^+^ production from CTLs, early effector CTLs, and predominantly central memory, T-helper/Memory cells expressing similar cytokines. It is possible that the adjuvant Poly(I:C) used in Nano-11-KAg delivered intranasal vs Amphigen a stable oil-in-water emulsion used in parenteral delivered commercial vaccine are playing critical role/s in these outcomes. Even though there is a predominance of IL-17A secreting cells in commercial vaccinates, it is possible that Th17 cells are transdifferentiating to Th1 cells generating IFNγ (36, 37), Alternatively, it is possible that IL-17A could be playing a dual role in commercial vaccine administered animals.

The *in vitro* generated IL-17A producing CTLs showed their ability to display memory phenotype and serve as a source of Type 1 CTLs secreting IFNγ and possess potent cytotoxic ability in mice (38). In this study, we characterized IL-17A production from both antigen specific CTLs and T-helper/Memory cells. There are several reports documenting an increased population of IL-17A producing T cells and secretion of IL-17A in pigs, such as the presence of *Actinobacillus pleuropneumoniae* specific Th17 cells [CD4^+^CD8α^dim^IL-17A^+^] in the blood and lungs of porcine contagious pleuropneumonia infected pigs (39). IL-17 is a proinflammatory cytokine, likely associated with inflammation in the lungs of pigs infected with Porcine Reproductive and Respiratory Syndrome Virus (PRRSV), because in the serum and BAL fluid of highly pathogenic PRRSV infected pigs IL-17A was detected, and in PRRSV infected alveolar macrophages *in vitro* high levels of IL-17A gene expression was observed (40). The presence of enhanced IL-17A producing cells in PBMCs was detected following PMA and ionomycin activation *in vitro* (41).

Furthermore, there was a report on switching of systemic Th1 to tissue-resident Th17 protective response upon mucosal administration of adjuvanted tuberculosis vaccine in mice and this protection was independent of TNFα and IL-17 receptors (42). Likewise, HIVgp140 adjuvanted with glucopyranosyl lipid adjuvant [a synthetic TLR-4 agonist] administered intranasally induced protective Th17 responses in mice (43). However, the administration of a detergent split influenza antigen adjuvanted with a TLR4 ligand CRX-601 intranasally led to the induction of harmful antigen specific CD4^+^TNFα^+^Th17^+^ cells in mice (44). Another group of researchers (45) reported the negative regulation of Th17 responses by IL-10 during influenza infection in mice and this effect correlates with defective protection in high dose primary but not secondary challenge infection. These results highlight the complex regulatory roles of IL-17A during infection. Further investigations are needed to elucidate the mechanisms governing the protective, inhibitory and pathological roles mediated by IL-17A during influenza infection so that better vaccine designs can be engineered to offer potent cross-protection against evolving field strains.

Our results are consistent with the published literature highlighting the critical role of antigen specific CTLs and T-helper/Memory cell responses against SwIAV (25, 33, 46). Furthermore, there are several reports that underline the pivotal roles of cell-mediated immune responses in a variety of settings. In pigs, the significant roles of CD4^+^ T-cells, CTLs and γδ T-cells have been demonstrated in PRRSV infection in pigs (47); CD8^+^ T-cells and T-helper/memory cells following pseudorabies live attenuated vaccination (48); CD3^+^ CD4^-^CD8α^hi^ CTLs in classical swine fever live attenuated vaccine and virulent challenge infection (49); and CD4^+^CD8α^hi^ αβ T-cells during melanoma tumor regression in swine model (50). Furthermore, Dengue virus-specific T-cell response in humans in response to live attenuated vaccination and challenge infection was detected (51). These findings emphasize the significance of cross-protective cell-mediated immune responses against conserved epitopes.

The commercial inactivated SwIAV vaccine is multivalent and consists of H1N1, H1N2, and H3N2 SwIAVs. The HA gene of our challenge virus H1N1-OH7 shares 95.2% and 77.9% identity with the corresponding H1N1 and H1N2, respectively, of the commercial vaccine. However, our monovalent Nano-11-KAg vaccine comprises of H1N2-OH10, which has 77% HA identity with the challenge H1N1-OH7 IAV. Hence, it is plausible that the commercial vaccine stimulated immune responses against the homologous virus H1N1; in contrast, the Nano-11-KAg+Poly(I:C) vaccine elicited cross-reactive cognate cell-mediated immune responses, associated with robust mucosal humoral and cytokine responses in MDA-positive pigs (**Supplementary Figure 3**). The control KAg+Poly(I:C) group exhibited significant antibody responses, which are consistent with a previous study in pigs (14), which can be attributed to the adjuvant Poly(I:C). In this study, we used KAg with Poly(I:C) co-adsorbed on Nano-11 to target the dendritic cells to ensure efficient induction of cognate cell-mediated immune responses conferring protection in the presence of MDA.

Designing nanovaccines coupled with a potent inducer of nonspecific effector responses of innate immune cells (trained immunity) (52), which elicit broad-spectrum protective immune responses targeting conserved internal T-cell and B-cell epitopes of viruses is an efficient strategy to combat constantly evolving and emerging IAV strains. Furthermore, unbiased systems-wide characterization of novel innate and adaptive correlates of protection are essential. Studies are underway to further improve the efficacy of the Nano-11-KAg vaccine by incorporation of multivalent SwIAV and split virus antigens coupled with potent adjuvant/s to further enhance the breadth of immunity, which is long-lasting against constantly emerging field strains of SwIAVs.

## Conclusions

The three IAV subtypes (H1N1, H1N2, and H3N2) circulating in the swine population are also prevalent among human seasonal flu viruses. The flu vaccines capable of eliciting broadly reactive humoral and cell-mediated immune responses in the respiratory tract are essential to mitigate both swine herds and human populations. Furthermore, to protect finisher pigs from SwIAV infections, a potent intranasal vaccine, which overcomes the interference caused by highly variable levels of MDA is required. Commercial multivalent SwIAV vaccine is capable of eliciting robust cellular immune responses but appears to be of a strong inflammatory nature, associated with the accumulation of naïve T-helper cells in both the mucosal lymph nodes and systemic compartment suggesting its possible inefficient antigen presentation nature in MDA-positive pigs. In contrast, the monovalent Nano-11-KAg+Poly(I:C) vaccine stimulated robust cross-reactive IgG and SIgA antibodies in the lungs and broadly protective polyfunctional cell-mediated immune responses to highly variant SwIAV, associated with a reduction in viral load in the airways and amelioration of lung pathology comparable to a multivalent commercial vaccine (**Supplementary Figure 3**). Thus, our study suggests the promise of intranasal delivery of Nano-11 and Poly(I:C) based inactivated SwIAVs vaccine in enhancing the breadth of cross-protective immunity against constantly emerging field strains of viruses in MDA-positive pigs, which will reduce economic losses from flu outbreaks.

## Data Availability Statement

The raw data supporting the conclusions of this article will be made available by the authors, without undue reservation.

## Ethics Statement

The animal study was reviewed and approved by Institutional Animal Care and Use Committee at The Ohio State University.

## Author Contributions

VP, SR, and GR conceived and developed the research, and VP and GR wrote the manuscript. SR formulated and characterized the vaccines. VP, SR, and NF-R did the experiments and analyzed the data. VP, SR, NF-R, JS, YH, and AR helped in vaccination and challenge trials in pigs, including sample collection and processing. VP performed flow cytometry analyses. HH analyzed the data and edited the manuscript. SD grew the vaccine and challenge viruses. All authors contributed to the article and approved the submitted version.

## Funding

This work was partially supported by the National Pork Checkoff. Salaries and research support were provided by state and federal funds appropriated to OARDC, The Ohio State University.

## Conflict of Interest

The authors declare that the research was conducted in the absence of any commercial or financial relationships that could be construed as a potential conflict of interest.
