# Supplementary figures and images for: Intranasal Delivery of Inactivated Influenza Virus and Poly(I:C) Adsorbed Corn-Based Nanoparticle Vaccine Elicited Robust Antigen-Specific Cell-Mediated Immune Responses in Maternal Antibody Positive Nursery Pigs
Source: Front Immunol. 2020 Dec 16;11:596964. doi: 10.3389/fimmu.2020.596964 (PMC7772411; doi:10.3389/fimmu.2020.596964)

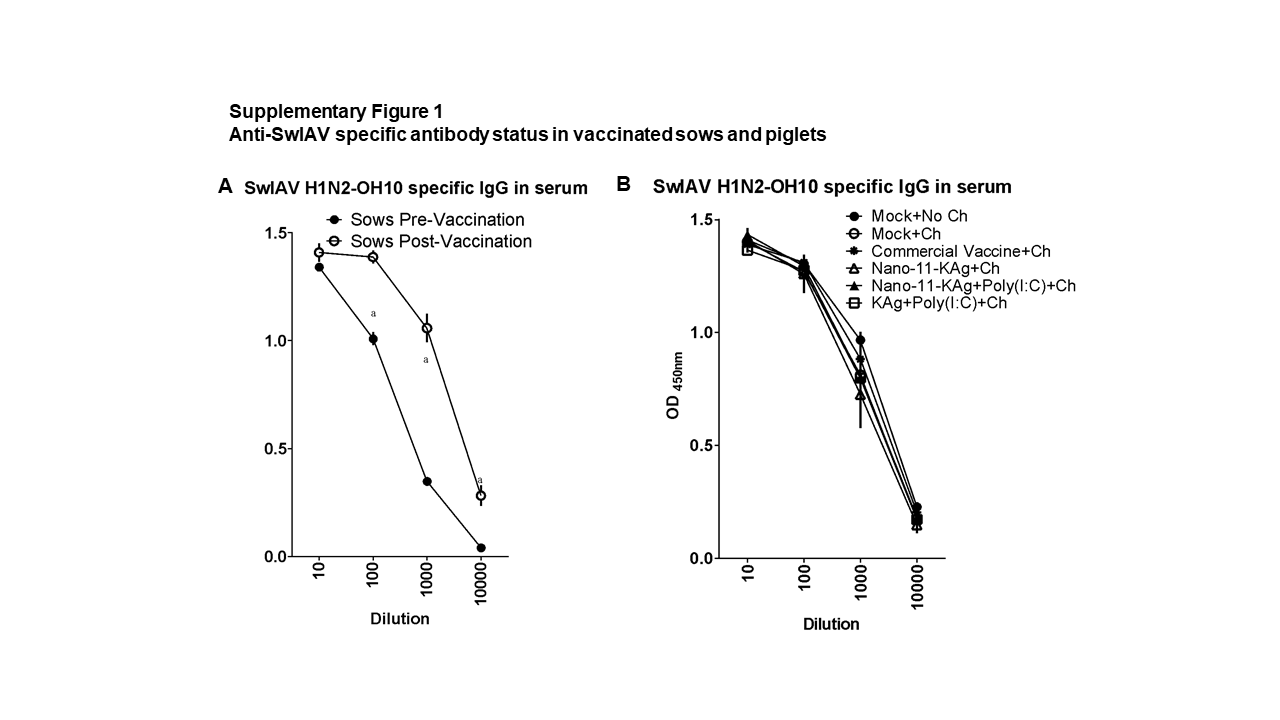

Supplement: Supplementary file 1 [file Image_1.tif]

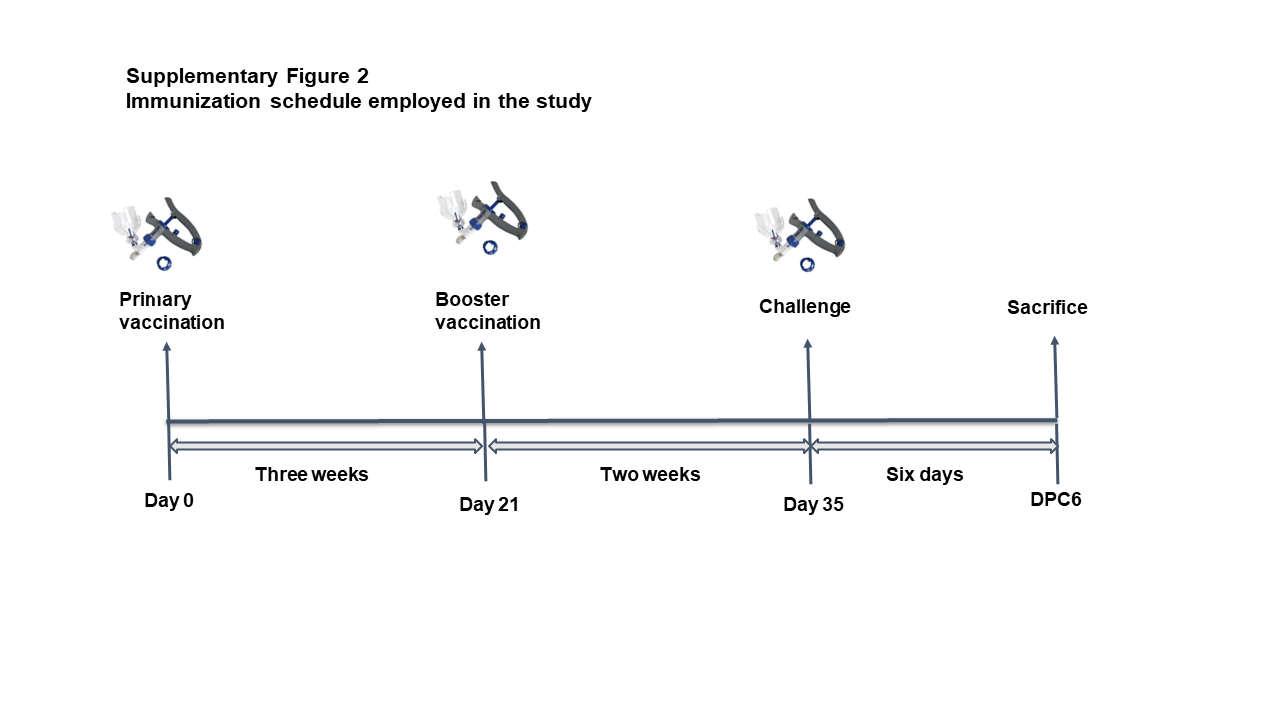

Supplement: Supplementary file 2 [file Image_2.tif]

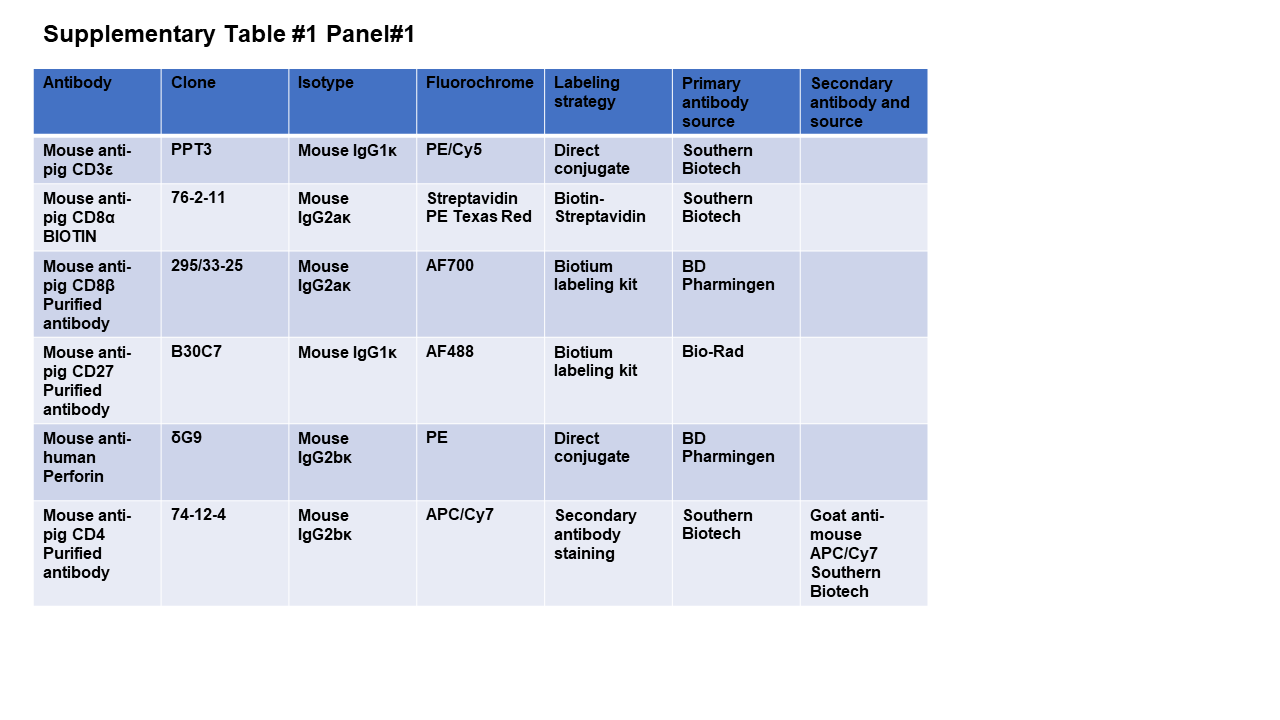

Supplement: Supplementary file 3 [file Image_3.tif]

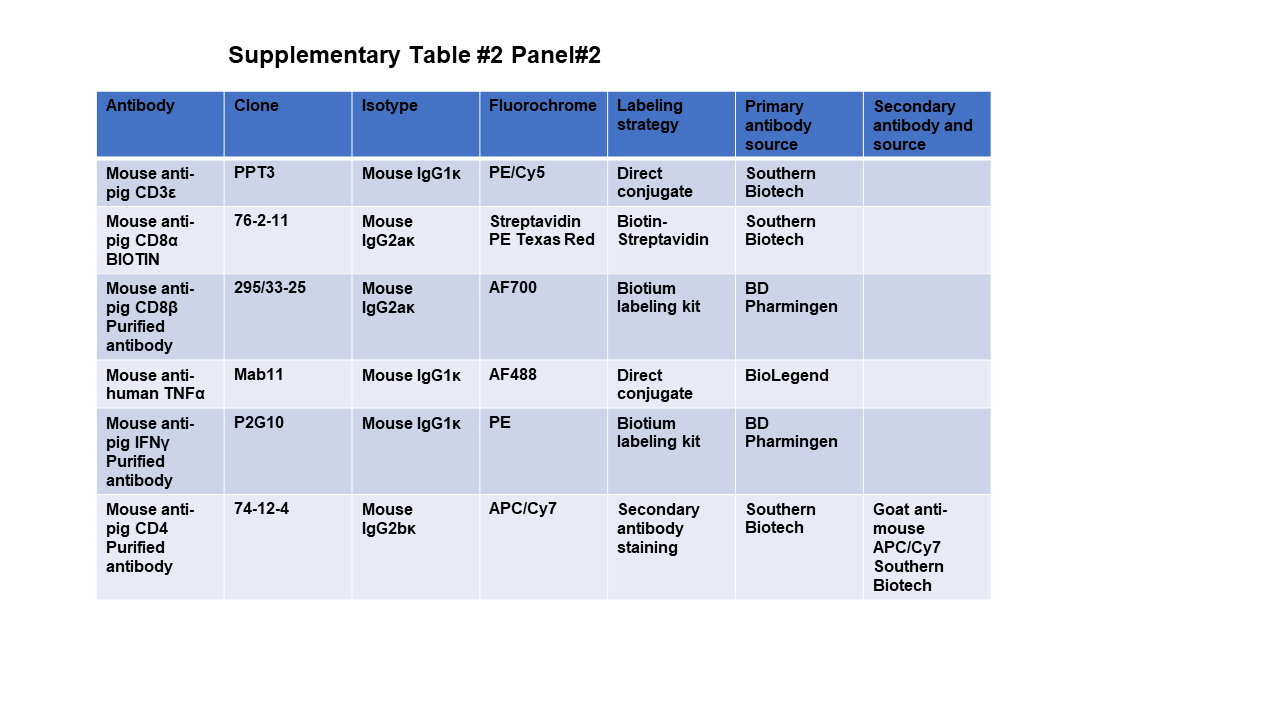

Supplement: Supplementary file 4 [file Image_4.tif]

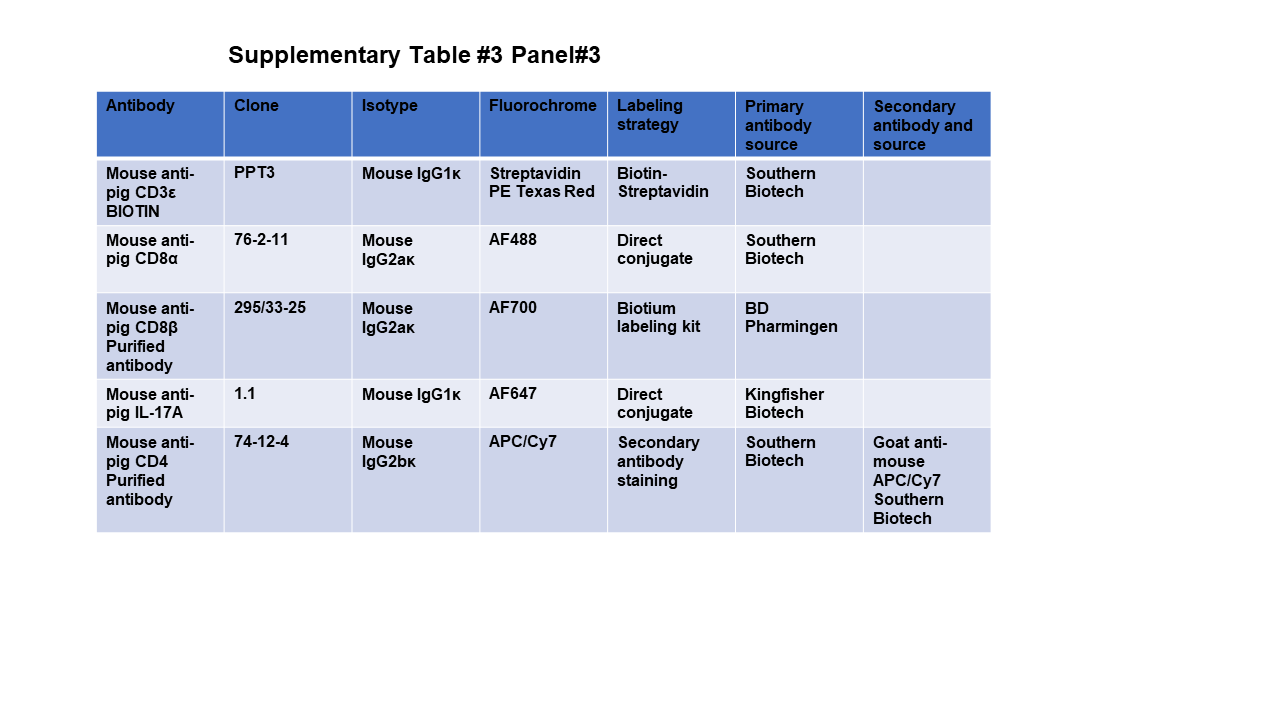

Supplement: Supplementary file 5 [file Image_5.tif]

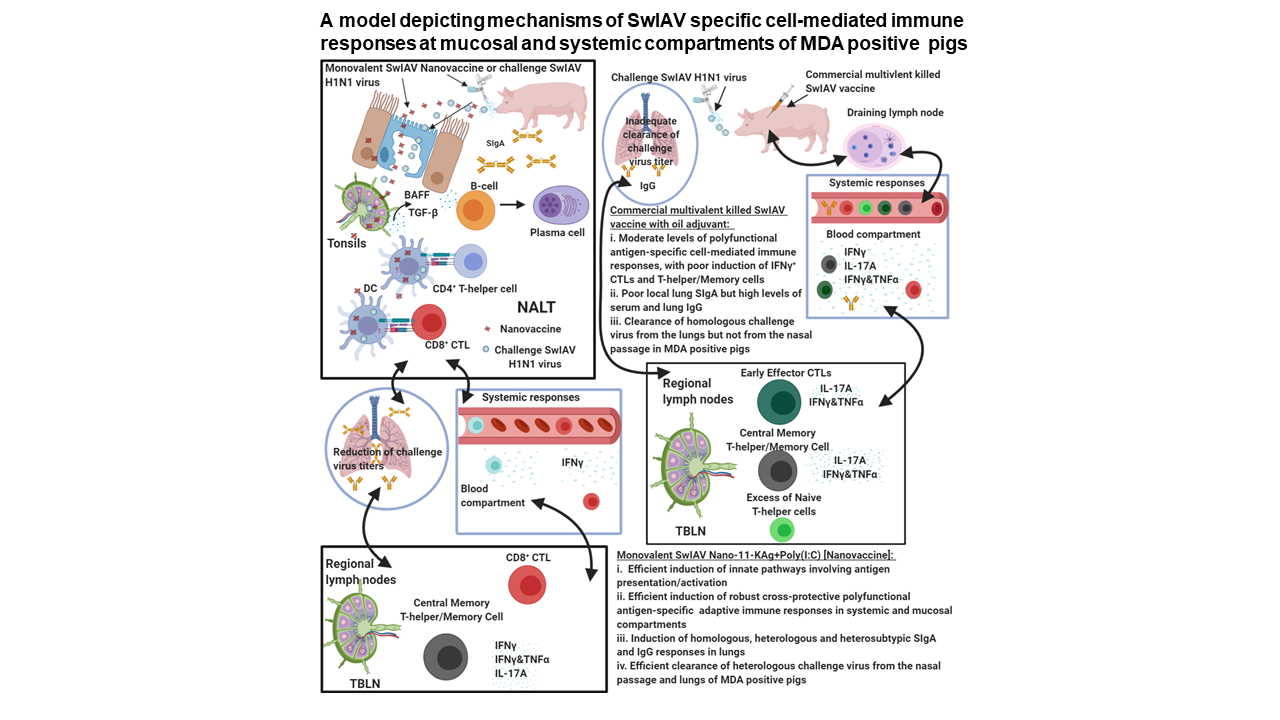

Supplement: Supplementary file 6 [file Image_6.tif]
